# Indoor air quality and the risk of hypertension

**DOI:** 10.1111/jch.14535

**Published:** 2022-07-29

**Authors:** Mihály Tapolyai, László Krivanek, Tibor Fülöp

**Affiliations:** ^1^ Department of Nephrology Szent Margit Hospital Budapest Hungary; ^2^ Medicine Services Ralph H. Johnson VA Medical Center Charleston South Carolina USA; ^3^ Szent Kristóf Szakrendelő Közhasznú Nonprofit Kft. Budapest Hungary; ^4^ Department of Medicine ‐ Division of Nephrology Medical University of South Carolina Charleston South Carolina USA

**Keywords:** air quality, combustion exposure, fine particulate matter, PM2.5, social determinants of health

1

The overwhelming majority of studies dealing with the connection between diet and hypertension up till today have focused on what we eat, not on how we prepare what we eat. To that end, the most recent publication of Peng et al.,^1^ derived from 10,400 participants in the China Health and Nutrition Survey provides additional clarification on the environmental risk profile of otherwise “essential” hypertension. The manuscript describes the results of a large, prospective nation‐wide representative cohort from across China with careful design and long follow‐up spanning over several decades, on the impact of combustion exposure for development of hypertension. The underlying tenet of the study is that cooking fire may elute substances that will contribute to the development of high blood pressure. The risk of new‐onset hypertension was adjusted for numerous variables during Cox regressions, including age, sex, BMI, ethnicity, education levels, marital status, gross family income, current smoking, alcohol consumption, physical activity, history of diabetes and cardiovascular diseases, dietary food choices, and stratified according to area of residence. Careful sensitivity analysis included adjustment for cooking fuel as a time‐dependent variable. They found that burning solid fuels such as coal, charcoal or “wood and sticks” may be harmful, representing a 58% increase in adjusted additive risk. Solid fuel burning in general posed an approximately 31% of increased risk when compared with use of gas/electricity. Even more alarmingly, their analysis suggested the biomedical sign was significant only in younger subjects, aged 18–39, and persistent independent of the time of enrollment into the study. Thus, the Authors authenticated an important moral: the risk of new‐onset hypertension with cooking fire exposure was more likely to impact younger people. Hypertension is, however, not just an elevation of blood pressure, but is likely to imply accelerated cardiovascular and renal outcomes, if with further delay. Older people, on the other hand, are more likely to have multiple co‐morbidities emerging with aging, competing for the risk of hypertension and detectable biomedical signal. While the Authors’ working theory that the risk of hypertension is caused by the fine particulate matter of aerodynamic diameter <2.5 ​μ (PM2.5), they do not elaborate on this potential pathological disease‐initiation; they did not measure or provide evidence for this. Notwithstanding, the aim of their study was not to explain the phenomenon but simply to show the process, and for this reason, it is not a true shortcoming of the study.

Fine‐particle exposure is known to generate inflammation^2^ and a recognized risk factor for multiple cardiovascular disease processes in industrialized societies, including of stroke,^3^ coronary artery calcifications,^4^ all‐cause cardiovascular disease,^5^ and declining renal function.^6^ Other adverse health outcomes in humans include new‐onset weight gain and obesity^7^ and neurotoxicity with impaired brain development.^8^ The mechanism through which smoking or even passive smoking may be deleterious in some health issues could be explained not only by the direct effect of gases or nicotine^9^ but the particulate matter emission while cigarettes burn.^10^, ^11^ On the contrary, improving indoor air quality may represent new avenues to improve BP control and reduce risk of hypertension across society.^12^

Also, it is fair assume that people who use polluting fuels belong to a poorer, less well‐to‐do social class? Socioeconomic status generally tracks with fine‐particle exposure burden,^13^ and may represent a practical translation of economic injustice into worse health outcomes. Adjustment for education, income and dietary choices were likely to remove most social determinants of the perceived health, but membership in hierarchal societal structures may still be present and unaccounted for. Perhaps the use of various cooking fuels may be a marker of social standing and social standing may have a bearing on the development of hypertension as seen among people of lower social classes.^14^ Social standing or the subjective perception of one's own social standing may also have a bearing on the development of cardiovascular risk factors. Even the subjective perception of social status may be associated with blood pressure elevations^15^ and the psychological response to one's status such as brooding and rumination can be a cardiovascular factor.^16^

Further inherent limitations and potential weaknesses of this study should be recognized, as well. As the authors have rightfully pointed out, this current study may have underestimated the PM2.5 exposure and burden as the association with cooking technology was listed exclusively; it is likely that these results are further impacted by heating choice, which was not listed and examined. Subjects using wood‐ or coal‐fueled stoves for cooking are likely to use the same stoves for heating rooms during cold seasons. In addition to particulate matter (PM2.5 and PM10), exposure to sulfur dioxide, nitrogen dioxide, and carbon monoxide may represent additional hazards. Environmental pollution and PM2.5 were not directly measured in the study's long duration, but adjusting for living area may have mitigated some of the confounders. We also noted that radon was not mentioned in the paper even though it conceivably may be present in coal at high concentrations but not in wood as cooking fuel. Radon has already been implicated in the development of hypertension in pregnant women and others.^17^ Measuring indoor radon in China, however, may have posed such a level of difficulty that would have halted the study.

In the recent years, these reviewers have witnessed important papers emerging from China further clarifying important aspects of hypertension risk. Examples include the effect of ambient temperature during pregnancy on the children's risk for subsequent hypertension^18^ or on the differential impact of centrally measured blood pressure to improve prediction of CV diseases.^19^ Studies with >10,000 participants and spanning over multiple decades represent a large societal commitment to preserve and improve health and represent enduring investment into preserving human capital. We wish to express our sincere appreciation of the study organizers, supporting institutions and agencies to enable the addition of this important paper to the world literature on hypertension. The current paper by Peng et al.^1^ is significant and provides a meaningful input on the potential attributable risk of indoor air pollution on the risk of hypertension. It has been said that we become what we eat; alas, it seems preparations do make a difference.

## CONFLICTS OF INTEREST

The authors alone are responsible for the content and writing of the paper. Dr. Tapolyai is a former employee of Fresenius Medical Care (FMC) Hungary. Drs. Tapolyai and Fülöp are current employees of the United States Veterans Health Administration. However, the opinions and views expressed in this paper are the Authors’ own and may not represent the official views or policies of either FMC Hungary, the Medical University of South Carolina or the United States Veteran Health Administrations. This paper has not received any financial support, endorsement, or oversight from any commercial entity.
